# Epidemiology of Falls Among Older Adults in Portugal: Analysis of Unintentional Injuries Reported by a National Emergency Surveillance System

**DOI:** 10.3390/healthcare13101160

**Published:** 2025-05-16

**Authors:** Tatiana Alves, Susana Silva, Paula Braz, Maria Papadakaki, Carlos Aniceto, Ricardo Mexia, Carlos Matias-Dias

**Affiliations:** 1 Epidemiology Department, National Institute of Health Doctor Ricardo Jorge, 1649 Lisbon, Portugal; susana.pereira@insa.min-saude.pt (S.S.); paula.braz@insa.min-saude.pt (P.B.); carlos.aniceto@insa.min-saude.pt (C.A.); ricardo.mexia@insa.min-saude.pt (R.M.); carlos.dias@insa.min-saude.pt (C.M.-D.); 2 Higher School of Health, Polytechnic Institute, 2914 Setubal, Portugal; 3 Department of Social Work, Hellenic Mediterranean University, 71004 Heraklion, Greece; mpapadakaki@hmu.gr

**Keywords:** injuries, home and leisure time, emergency department, hospitals, Portugal

## Abstract

**Background/Objectives**: Falls occurring at home and during leisure time among elderly individuals represent a serious public health issue in Portugal and worldwide. These incidents have a significant impact on healthcare systems and social support structures, as well as the personal and family lives of the victims. There is also a recognized gap in awareness among older adults regarding fall prevention, particularly regarding environmental hazards, the need for home modifications, and the adoption of safety behaviors, including necessary adjustments in their home environments. The present study was developed to enhance our understanding of the circumstances in which falling occurs in elderly people. **Methods**: A cross-sectional epidemiological study was carried out, analyzing data collected through the national emergency-based injury surveillance system in 2023. **Results**: The proportion of falls increased across age groups, with 34.9% of total falls occurring in the group aged 85 and over. These differences were statistically significant (*p* < 0.001). In all age groups, falls were more frequent among women, representing between 63.6% and 69.0% of episodes. Approximately 65.9% of falls occurred at home. The likelihood of falling was higher among the oldest age group (85+) and in the home. **Conclusions**: The results of this study confirm that falls in elderly people tend to occur more frequently with advancing age, particularly in environments where the most time is spent. This study suggests that fall-prevention campaigns should be specifically targeted towards older females in the home environment, with particular consideration given to morning routines as part of the recommended interventions.

## 1. Introduction

Falls are a global public health concern and represent the second leading cause of unintentional injury-related deaths, following road traffic accidents [1,2]. While falls can occur across all age groups, age is a well-established risk factor [3]. Understanding falls among older adults is particularly important within the global context of population ageing.

Falls are also a critical issue for emergency department (ED) teams, as they represent the leading cause of external injuries and account for approximately 24% of injury-related ED visits [4].

Numerous studies have shown that the frequency of falls increases with age and frailty. The risk of falling increases progressively in both men and women, across rural and urban populations, and among all ethnic groups [1].

Approximately 28–35% of individuals over the age of 65 experience at least one fall per year, with about half of them falling more frequently. Among those older than 70 and living in the community, the frequency increases to 32–42% [1].

Different factors have been identified in the occurrence of falls, usually classified as intrinsic and extrinsic. The intrinsic factors are usually related to chronic diseases, associated health conditions, sleep disorders, and polypharmacy. Due to their modifiable nature, environmental hazards—classified as extrinsic factors—are involved in 33–50% of falls [4]. Generally, it has been documented that most falls in the elderly occur indoors, namely, in the bathroom, bedroom, and kitchen. Elderly people tend to fall at home during routine activities.

In Portugal, demographic ageing is a well-documented reality, and this trend is expected to continue. This is driven not only by a decrease in the young and working-age population, but also by a steady increase in the number of older adults [5]. As a result, the incidence of falls are projected to increase, not only due to the growing elderly population, but also due to the age-related burden of comorbidities, polypharmacy, and frailty [4,6]. This evolving scenario could lead to a greater demand for emergency services [2].

According to data from the national injury surveillance system (EVITA), falls account for over 70% of injury mechanisms in home- and leisure-related incidents among older adults who present to emergency departments [7]. Furthermore, national data from 2023 show that approximately 11% of people aged 65 and older sought help in emergency departments due to a fall [8].

Several studies have highlighted the importance of fall prevention in older people, due to the significant impact falls can have on motor function, mobility, autonomy, and self-confidence [1,3]. These consequences often lead to mental health challenges, such as depression [6,7], anxiety [6,7], and social isolation [4].

Falls among older adults are often described in the literature as a multifactorial phenomenon, involving both intrinsic and extrinsic factors. The living environment plays a crucial role, as the characteristics and dynamics of falls vary depending on the context and location—particularly within the home or residential care settings [4,9].

Different factors have been reported to contribute to the occurrence of falls, which are usually classified as intrinsic and extrinsic. Intrinsic factors commonly include chronic diseases, comorbid health conditions, sleep disorders, and polypharmacy. On the other hand, extrinsic factors, such as environmental hazards, are modifiable and have been found to contribute to 33–50% of falls. These modifiable risks are often targeted in fall-prevention programs [4]. Research consistently shows that most falls among older adults occur indoors, especially in bathrooms, bedrooms, and kitchens, usually during routine daily activities.

Maintaining good health, autonomy, and independence for as long as possible is a major challenge for individuals and society alike, and has significant implications for national economic development. Nevertheless, the reality is that the later years of life are often marked by frailty and disability, conditions that are often preventable, especially in the case of falls.

Nurses play a critical role in fall prevention within the community setting. Although various nursing interventions have been developed to address this public health concern, understanding fall characteristics in specific contexts can support more tailored and effective strategies [10,11].

The aim of this study is to describe the characteristics of same-level and stair-related falls among individuals aged 65 and older, who accessed emergency services within Portugal’s National Health Service in 2023. The study seeks to increase knowledge regarding the factors associated with falls in this population.

## 2. Materials and Methods

An observational, descriptive, cross-sectional epidemiological study was conducted, analyzing data from the national injury surveillance system (EVITA) for hospital emergency episodes in 2023 involving individuals aged 65 years and older in Portugal [12].

The EVITA system is a health observation tool that enables the monitoring of home and leisure accidents (HLA). The data were extracted from the Sistema Integrado de Informação Hospitalar (SONHO), the Integrated Hospital Information System responsible for generating, controlling, and invoicing administrative and clinical data, including patient history, diagnoses, and benefit information. SONHO facilitates the export of data for statistical indicators and supports clinical data sharing across hospitals and health centers that use this system (http://im.med.up.pt/si_saude/si_saude.html; https://aprendis.med.up.pt/index.php/SONHO, accessed on 1 May 2025). This system is used in more than 90% of health institutions within Portugal’s National Health System (SNS), encompassing public and public−private partnership hospitals throughout all regions, ensuring extensive national coverage and representing the entire population.

A descriptive analysis (counts) was carried out based on variables including age group, sex, day of the week, time of the accident, place of occurrence, and discharge destination. Bivariate analysis was performed using Pearson’s chi-squared test with a significance level set at 5%. For the inferential analysis, a logistic regression model was used and the results were presented as adjusted odds ratios (ORs) with 95% confidence intervals. The analysis was performed using the R Statistical Computing Environment program (R version 4.1.2) [13].

## 3. Results

This study analyzed the 35,312 emergency department admissions resulting from same-level and stair-related falls among older adults, as recorded in the national injury surveillance system (EVITA). An overall increase in the proportion of falls was observed across all age groups, with 34.9% of all falls occurring in individuals aged 85 and older.

When examining fall episodes by age and sex, falls were more frequent among women in all age groups, accounting for between 63.6% and 69.0% of cases. These differences were statistically significant (*p* < 0.001) (Table 1).

The timing of falls varied throughout the day, with the majority occurring during the morning and afternoon, between 09:00 a.m. and 17:00 p.m. This timeframe accounted for 52.3% of falls in the 85 and older age group and 55.5% in the 75–79 age group. Sunday had the lowest number of fall-related emergency department admissions across all age groups, although no statistically significant association was found between the day of the week and the age group.

Falls occurred in a variety of settings, with the home being the most common location (22,535 cases; 65.9%). Notably, the proportion of falls occurring at home increased with age, from 64.4% in the 65–74 age group to 68.9% in the 75–84 years. In the 65–74 age group, the most frequent fall locations after the home were transport areas (14.6%) and outdoor spaces (11.4%). In the 85 and. older age group, the second most common location was institutional settings (24.9%), with more than 80% of these falls occurring in residential or nursing homes.

Regarding discharge outcomes, the proportion of fall-related cases resulting in hospital admission increased with age, from 8.7% in the 65–74 age group to 18.7% in those aged 85 and over. Conversely, the proportion of cases that did not require further medical consultation, either in primary care or in hospital, decreased with age, from 49.0% in the 65–74 group to 43.4% in the 85 and over group.

To examine the association between fall occurrence and factors such as sex, age group and place of occurrence, a logistic regression model was applied.

**Figure 1:**
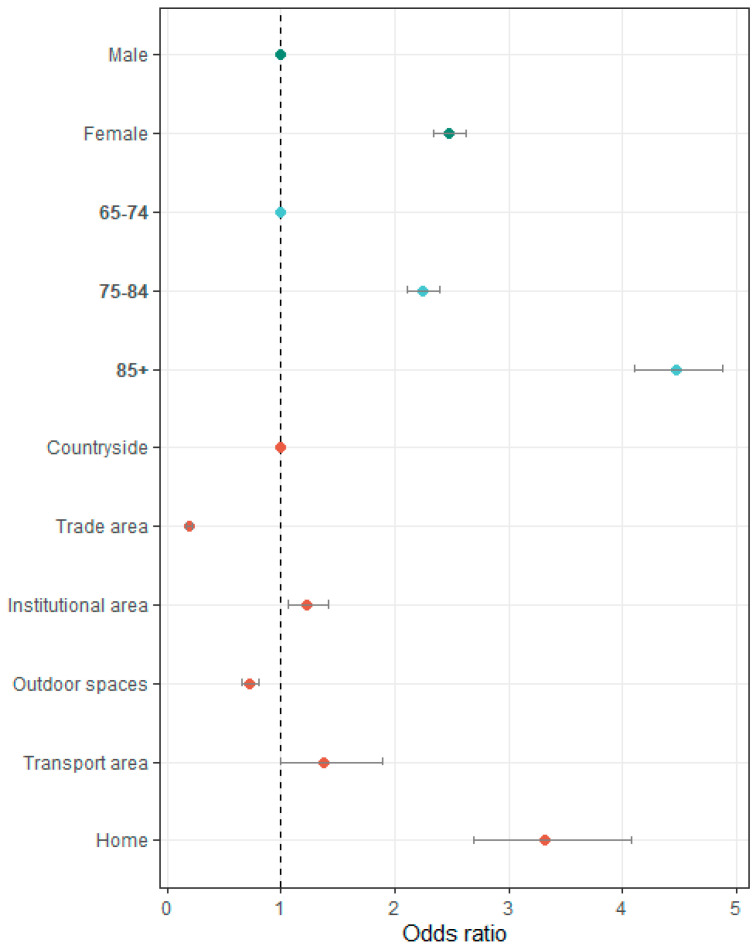
Adjusted odds ratio falls attended at the hospital emergency department, in the elderly people, in Portugal during the 2023 year by sex, age group and place of occurrence.

Regarding sex, the analysis showed that the odds of fall-related emergency department visits among older adults in Portugal during the 2023 year were 2.47 times higher in females than in males, a statistically significant finding (OR = 2.47; 95% [2.33, 2.62]) (Figure 1). In terms of age, individuals aged 85 and over had 4.47 times higher odds of experiencing a fall resulting in ED visit compared to those aged 65–74 year (OR = 4.47; 95% [4.10, 4.87]) a statistically significant result. Similarly, individuals in the 75–84 age group had higher odds of falls compared to the 65–64 age group (OR = 2.24, 95% [2.10, 2.38]).

With respect to the place of occurrence, the odds of experiencing a fall at home were 3.31 times higher than in countryside settings, a statistically significant difference (OR = 3.31, 95% [2.69, 4.07]). Additionally, falls occurring in institutional settings were 1.23 times more likely than those occurring in the countryside (OR = 1.23; 95% CI [1.06, 1.42]). In contrast, falls that occurred in outdoor spaces (OR = 0.72; 95% CI [0.65, 0.79]) and in commercial areas (OR = 0.19; 95% CI [0.17, 0.22]) were significantly less likely compared with falls in countryside settings.

## 4. Discussion

Falls among elderly individuals represent a significant public health concern, given their high frequency, impact on healthcare utilization, and their role as a leading cause of hospital emergency departments visits. 

In the population aged 65 years and over, falls emerged as the most common mechanism of injury. This study revealed an age-related increase in the proportion of falls, aligning with international findings that identify falls as the primary cause of emergency department admissions for unintentional injuries among the elderly [6,7,14,15,16,17,18].

The literature consistently underscores that while falls are a multifactorial phenomenon, age is one of the most frequently cited biological risk factors. Several studies have highlighted the role of aging in functional decline, leading to increased frailty and a higher likelihood of falling [3,4,16,17,18].

As observed in Portugal and other countries, women are more frequently affected by falls than men [16,19]. Various biological conditions associated with the female sex, including musculoskeletal disorders, are thought to contribute to this higher susceptibility to falls [4,10,17].

Regarding the timing of falls, this study found that they occurred most often toward the end of the week and during daily hours, which seems to be linked to the daily routines and activities of older adults. This pattern aligns with findings from other national and international studies [2,16,17]. However, a contrasting study conducted in Turkey reported no significant variation in fall incidence based on the time of day [7].

We found older adults requiring hospitalization as a result of a fall tended to be in the oldest age groups. This corroborates previous research indicating that increased age is associated with more severe injuries and longer hospital stays [4,20,21]. These findings highlight the need for health services, particularly nursing teams, in order to understand the implications of falls and plan interventions aimed at secondary and tertiary prevention in ageing populations.

Given their close interactions with patients, nurses—alongside other healthcare professionals such as physicians, pharmacists, physiotherapists, and occupational therapists—should play a key role in fall prevention. They need to be aware of the dimensions and characteristics of falls, recognize that older adults are at higher risk, and identify specific contributing factors. Working within an interdisciplinary team, nurses can contribute to implementing strategies and interventions aimed at modifying fall risk factors and improving safety in this vulnerable population [11,22]. Additionally, in the context of home hospitalization, nurses play a crucial role in educating older adults and their families about personalized measures to prevent falls. They consider the unique features of each residence and the individual’s clinical conditions, providing ongoing monitoring to ensure safety [23,24].

The regression analysis revealed that being female and of an advanced age were independently associated with an increased risk of falls. These results are in line with other studies indicating out that being female is a risk factor for falls [25]. In comparison with males, females typically have lower muscle mass and weaker musculoskeletal systems [26]. Our study is also aligned with other studies showing that older adults are at a substantially greater risk for falls compared with younger adults [2].

The setting in which falls occur have also been increasingly studied, and several studies have shown that falls in older individuals predominantly occur at home and in institutional settings, similar to our observations in this study. Conversely, fewer falls occur in outdoor or public areas, possibly due to decreased mobility and activity levels among older adults, which reduce their exposure to these environments. Nonetheless, the literature emphasizes the importance of assessing and eliminating hazards in all settings to reduce environmental risks that contribute to falls [4,9,16,18].

While acknowledging the contributions of this study, it is crucial to recognize the inherent limitations that may influence the interpretation of its findings. The analysis focused exclusively on falls among individuals aged 65 and older who received emergency treatment at hospitals within Portugal’s National Health Service. This approach may overlook less serious falls treated outside hospital settings or those that did not require medical attention, suggesting that the true incidence and impact of falls among older adults may be significantly underestimated. This raises questions regarding the thoroughness and precision of the surveillance data, as minor falls—despite being less severe—can still have significant consequences for health and well-being.

Moreover, the reliance on data collected from hospital admissions reported through the EVITA system, while methodologically standardized and in alignment with European injury surveillance protocols, limits generalizability to the broader older adult population. It presumes that documented cases are entirely representative; however, it overlooks individuals who fall and do not seek hospital treatment or those who receive care in other healthcare environments, resulting in an inherent selection bias. This underscores the need to interpret these findings within the context of the population’s healthcare-seeking patterns and the system’s ability to record all pertinent incidents.

On the other hand, a major strength of this study is its methodical data collection and adherence to a standardized European framework, which enhances the reliability and comparability of the findings across regions. This methodological rigor provides a strong foundation for guiding targeted interventions; however, the results should be viewed with an understanding of their limitations and potential gaps. Recognizing these limitations highlights the necessity for broader, multisource data collection methods to clarify the true extent of fall-related injuries in older adults, and to develop more effective preventative strategies.

## 5. Conclusions

The proportion of falls increased across all age groups. The likelihood of falling was higher among older individuals, women, those living at home, and residents in elderly care facilities.

As shown in the literature, there is a pressing need to deepen our understanding of the factors associated with falls in older adults, especially in the context of current demographic trends and population aging. Ongoing research in this area is essential, as it supports the development of targeted fall-prevention programs and informs preparedness across various levels of healthcare.

This study highlights the importance of addressing falls among older adults as a critical issue linked to population ageing. It also highlights the need for increased awareness and engagement among those who play a vital role in their lives, such as family members, caregivers, nurses, and other health professionals. In particular, this study suggests that fall-prevention campaigns should be targeted specifically towards older females in home settings, and recommendations including morning daily routines should be considered. Also, these findings contribute to fall surveillance systems by drawing attention to variables that need further study.

## Figures and Tables

**Table 1:** Distribution of fall episodes in people aged 65 and over, by sex, day of the week, time of accident, place of occurrence, and discharge destination (EVITA, 2023).

		Age Group					
	65–74		75–84		≥85		
	n	%	n	%	n	%	*p*-Value ^1^
**Total**	9916	28.1	13,076	37.0	12,320	34.9	
**Sex**							<0.001
Male	3610	36.4	4299	32.9	3825	31.0	
Female	6306	63.6	8777	67.1	8495	69.0	
**Week day**							0.392
Sunday	1356	13.7	1783	13.6	1689	13.7	
Monday	1383	13.9	1991	15.2	1839	14.9	
Tuesday	1418	14.3	1872	14.3	1750	14.2	
Wednesday	1423	14.4	1843	14.1	1812	14.7	
Thursday	1405	14.2	1851	14.2	1726	14.0	
Friday	1481	14.9	1834	14.0	1733	14.1	
Sunday	1450	14.6	1902	14.5	1771	14.4	
**Accident time**							<0.001
00:00–08:59	1230	12.4	1934	14.8	2413	19.6	
09:00–16:59	5444	54.9	7261	55.5	6440	52.3	
17:00–24:59	3242	32.7	3881	29.7	3467	28.1	
**Occurrence place**							<0.001
Home	6123	64.4	8695	68.9	7717	64.1	
Outdoor spaces	1083	11.4	1046	8.3	509	4.2	
Transport area	1387	14.6	1257	10.0	640	5.3	
Trade area	180	1.9	184	1.5	78	0.6	
Institutional area	415	4.4	1178	9.3	2998	24.9	
Country area	320	3.4	266	2.1	100	0.8	
**Destination after discharge**							<0.001
Abandonment	282	2.8	234	1.8	159	1.3	
Unreferenced exterior	4850	49.0	6068	46.5	5384	43.8	
Referenced	3496	35.3	4625	35.4	3946	32.1	
Hospitalization	857	8.7	1598	12.2	1598	18.7	
Transferred another hospital	221	2.2	279	2.1	257	2.1	
Decease	5	0.1	13	0.1	24	0.2	
Other	186	1.9	233	1.8	218	1.8	

^1^ Pearson’s Chi-square test.

## Data Availability

The original contributions presented in this study are included in the article. Further inquiries can be directed to the corresponding author.

## Abbreviations

The following abbreviations are used in this manuscript:

EVITA	Portugal injury surveillance system
HLA	Home and Leisure Accidents
SONHO	Integrated Hospital Information System
